# Ancient gene transfer from algae to animals: Mechanisms and evolutionary significance

**DOI:** 10.1186/1471-2148-12-83

**Published:** 2012-06-12

**Authors:** Ting Ni, Jipei Yue, Guiling Sun, Yong Zou, Jianfan Wen, Jinling Huang

**Affiliations:** 1 State Key Laboratory of Genetic Resources and Evolution, Kunming Institute of Zoology, Chinese Academy of Sciences, Kunming, Yunnan, 650223, China; 2 Graduate School of the Chinese Academy of Sciences, Beijing, 100039, China; 3 Department of Biology, East Carolina University, Greenville, NC, 27858, USA

**Keywords:** Gene transfer, Endosymbiosis, Plastids, Animal evolution

## Abstract

**Background:**

Horizontal gene transfer (HGT) is traditionally considered to be rare in multicellular eukaryotes such as animals. Recently, many genes of miscellaneous algal origins were discovered in choanoflagellates. Considering that choanoflagellates are the existing closest relatives of animals, we speculated that ancient HGT might have occurred in the unicellular ancestor of animals and affected the long-term evolution of animals.

**Results:**

Through genome screening, phylogenetic and domain analyses, we identified 14 gene families, including 92 genes, in the tunicate *Ciona intestinalis* that are likely derived from miscellaneous photosynthetic eukaryotes. Almost all of these gene families are distributed in diverse animals, suggesting that they were mostly acquired by the common ancestor of animals. Their miscellaneous origins also suggest that these genes are not derived from a particular algal endosymbiont. In addition, most genes identified in our analyses are functionally related to molecule transport, cellular regulation and methylation signaling, suggesting that the acquisition of these genes might have facilitated the intercellular communication in the ancestral animal.

**Conclusions:**

Our findings provide additional evidence that algal genes in aplastidic eukaryotes are not exclusively derived from historical plastids and thus important for interpreting the evolution of eukaryotic photosynthesis. Most importantly, our data represent the first evidence that more anciently acquired genes might exist in animals and that ancient HGT events have played an important role in animal evolution.

## Background

Gene transfer, including those occurred horizontally between distinct species (horizontal gene transfer, HGT) or those intracellularly from organelles (mitochondria or plastids) to the nucleus (endosymbiotic gene transfer, EGT), has now been widely recognized as an important force in organismal and genome evolution [1-3]. In particular, HGT may rapidly spread evolutionary success across lineages and allow recipient organisms to access new niches or other resources. Although numerous cases of HGT have been documented in prokaryotes and unicellular eukaryotes, a common belief is that HGT is rare in multicellular eukaryotes such as animals and plants [4,5]. Such a belief, however, largely ignores the dynamic nature of HGT and the more ancient HGT events to the unicellular ancestors of multicellular eukaryotes [6].

Gene transfer, although usually disruptive in phylogenetic reconstruction, may also serve as molecular fossils or footprints for historical evolutionary events [7,8]. An intriguing case in this respect is the distribution of algal/cyanobacterial genes in eukaryotes. Because eukaryotic photosynthesis is derived from a primary endosymbiosis with a cyanobacterium as well as secondary and tertiary endosymbioses with miscellaneous algae [9-12], these genes are frequently interpreted as relicts of earlier cyanobacterial/algal endosymbionts and, therefore, evidence of plastid losses in some aplastidic eukaryotes [13-15]. As eukaryotic photosynthesis spans multiple major lineages whose relationships critically rely on the plastid existence in them [16-18], an accurate understanding of the distribution and origin of algal genes in eukaryotes is essential.

Several recent studies indicated that algal genes also exist in organisms that are generally considered to be plastid-lacking throughout their evolution [15,19-21]. In particular, over 100 genes of algal origins were found in the choanoflagellate *Monosiga brevicollis*[19,21]. Because of the widespread distribution of algae and the phagotrophic nature of choanoflagellates, it was suggested that algal genes in *Monosiga* might have been acquired from food sources [21]. However, because choanoflagellates are the closest unicellular relatives of animals [22], it is unclear whether such acquisition of algal genes represents species-specific events or might have occurred also in the unicellular ancestor of animals and been inherited by diverse extant animals.

To address the above question, we performed phylogenomic analyses of the tunicate *Ciona intestinalis* to search for algal genes, in particular those anciently acquired by the ancestral animal. We choose *C. intestinalis* as a query to detect algal genes in animals because of its relatively small genome size and frequent physical contact with phytoplanktons. We here report 14 gene families of likely algal origin in *C. intestinalis* and other animals. We also further discuss the potential mechanisms of algal gene acquisition in animals and the importance of HGT in animal evolution.

## Results and discussion

### Algae-related genes identified in *C. intestinalis* and other animals

**Figure 1:**
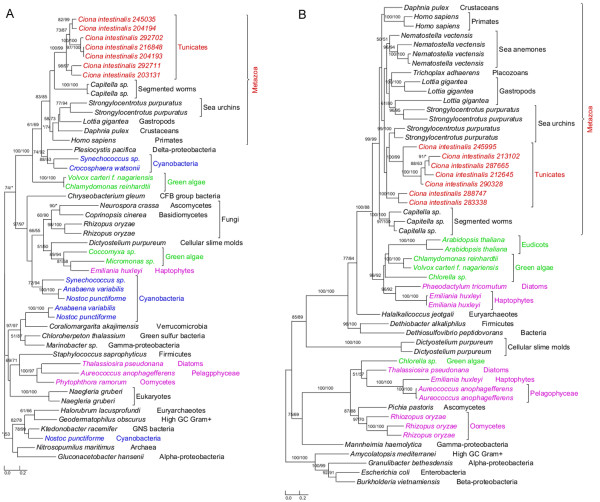
**Phylogeny of algal genes identified in *Ciona ****.* Numbers above branches show bootstrap values for maximum likelihood and distance analyses respectively. Asterisks indicate values lower than 50%. Other bootstrap values below 50% in both methods are not shown. Red: tunicates; green: Plantae; blue: cyanobacteria; pink: other plastid-containing eukaryotes. (**A**) Short-chain dehydrogenase/reductase family and (**B**) solute carrier family 23.

**Figure 2:**
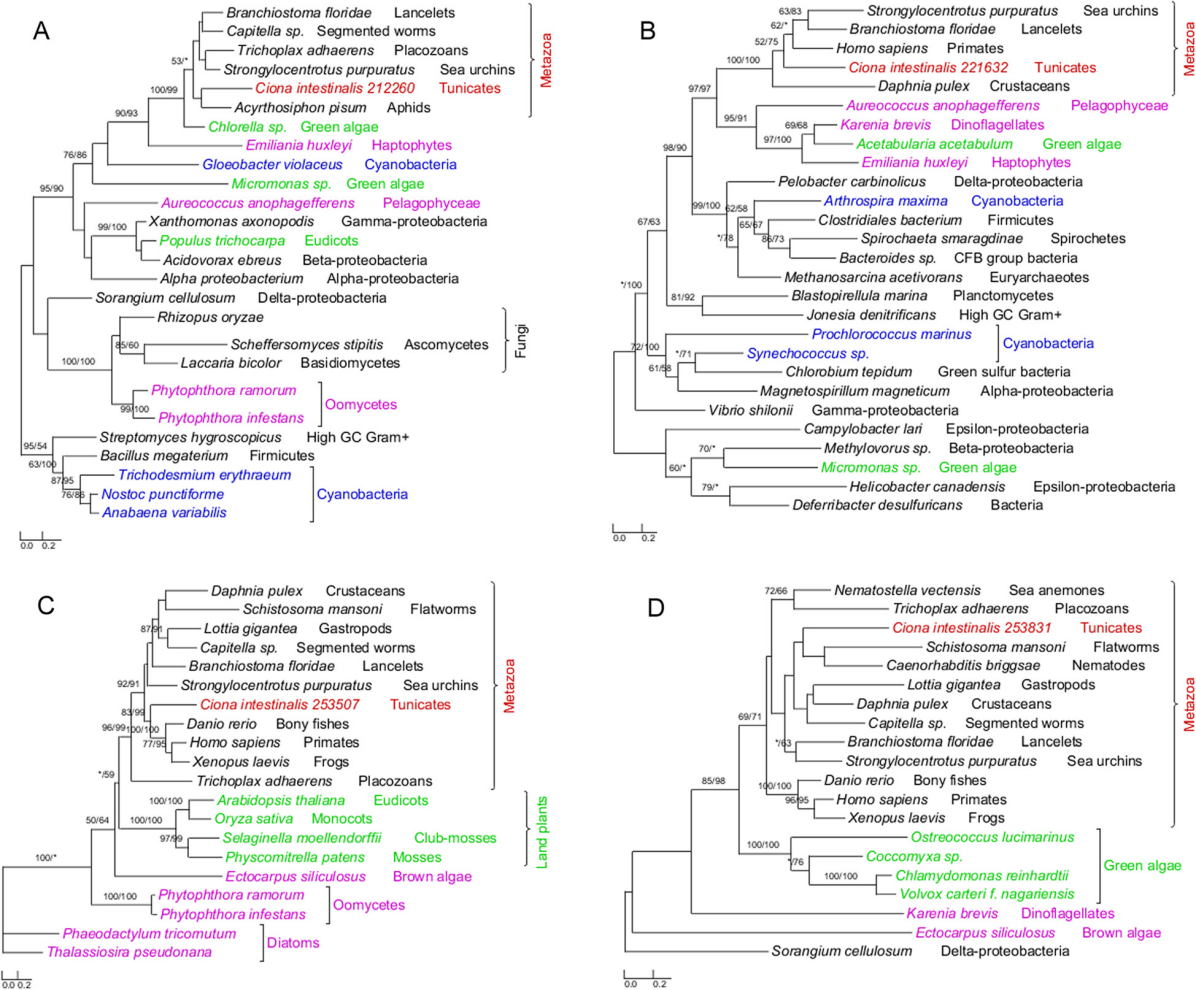
**Phylogeny of algal genes identified in *Ciona. *** Numbers above branches show bootstrap values for maximum likelihood and distance analyses respectively. Asterisks indicate values lower than 50%. Other bootstrap values below 50% in both methods are not shown. Red: tunicates; green: Plantae; blue: cyanobacteria; pink: other plastid-containing eukaryotes. (**A**) Taurine dioxygenase; (**B**) cytidine monophospho-N-acetylneuraminic acid synthetase; (**C**) arginine N-methyltransferase 7; and (**D**) peptidylglycine alpha-hydroxylating monooxygenase.

**Figure 3:**
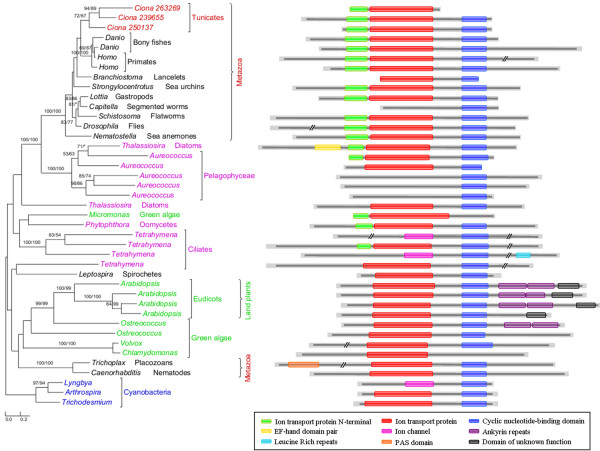
**Phylogenetic and Pfam domain analyses of potassium/sodium hyperpolarization-activated cyclic nucleotide-gated channel 2.** Numbers above branches show bootstrap values for maximum likelihood and distance analyses respectively. Asterisks indicate values lower than 50%. Other bootstrap values below 50% in both methods are not shown. Red: tunicates; green: Plantae; blue: cyanobacteria; pink: other plastid-containing eukaryotes. Three identical tandem domains (Ion transport protein N-terminal/Ion transport protein/Cyclic nucleotide-binding domain) are restricted to animals and algae. Several animal and algal sequences contain only one or two domains, suggestive of potential loss over time.

The annotated genome of *C. intestinalis* consists of 14,002 protein-coding genes and genome screening using *AlienG*[23] identified 169 of them as candidates of algal origin. We performed detailed analyses of taxonomic distribution, gene structure and molecular phylogeny for each of the identified candidate genes. One of these genes (GenBank accession number: XP_002127943) has identifiable homologs only in animals and plastid-containing eukaryotes. Another identified gene (GenBank accession number: XP_002122576) is restricted to animals, plastid-containing eukaryotes and ciliates, which also contain algal genes because of their possible historical plastids or phagotrophic lifestyle [14]. For additional 90 genes, although their molecular phylogenies often lack sufficient resolution in some branches, algal and animal sequences form monophyletic groups with over 70% bootstrap support (Figures 1,2 and 3). Therefore, 92 of the 169 genes are considered to be algae-related. Additional evidence from Pfam domain analyses [24] also indicates animal/algae affinity. For instance, three genes encoding potassium/sodium hyperpolarization-activated cyclic nucleotide-gated channel 2 contain three identical tandem domains only in animals and algae (Figure 3). These 92 genes can be further classified into 14 gene families or subfamilies, including those encoding solute carriers, short-chain dehydrogenase/reductase, and UDP galactopyranose mutase etc (Table 1), based on their sequence relatedness (30-80% protein sequence identities and similar tree topologies) and GO annotation information (Additional file 1). Except the genes encoding UDP galactopyranose mutase, all other algae-related genes are present in multiple groups of animals (Figures 1,2 and 3; Additional file 2)*.*

**Table 1:** **Putative algal genes identified in *Ciona intestinalis ***

**Gene family**^**a**^	**Putative donor**	**Putative function**
Short-chain dehydrogenase/reductase family (7)	Green algae/ cyanobacteria	Cellular differentiation and signaling
Solute carrier family 23 (7)	Algae/land plants	Na^+^-dependent vitamin C transport
Taurine dioxygenase (1)	Green algae	Cell redox homeostasis
Cytidine monophospho-N-acetylneuraminic acid synthetase (1)	Algae	Signaling regulation
Arginine N-methyltransferase 7 (1)	Land plants/ stramenopiles	Protein methylation
Peptidylglycine alpha-hydroxylating monooxygenase (1)	Green algae	Hormone biosynthesis
Aminoglycoside phosphotransferase (1)	Algae	Protein amino acid phosphorylation
Solute carrier family 22 (27)	Algae/land plants	Ion transport
Solute carrier family 34 (6)	Diatoms	Phosphate ion transport
Potassium/sodium hyperpolarization-activated cyclic nucleotide-gated channel 2 (3)	Stramenopiles	Potassium ion transport
UDP galactopyranose mutase (2)*	Algae	Uridine diphosphogalactofuranose biosynthesis
Biphenyl/valacyclovir hydrolase (3)	Dinoflagellates/haptophytes	Aromatic compound metabolism
Solute carrier family 6 (31)	Algae	Neurotransmitter transport
Alkylation repair homolog 5 (1)	Algae/land plants	Alkylation repair

^a^Numbers in the brackets show paralogous gene copies within each family. *The gene transfer event happened only between algae and *C. intestinalis*. All other gene transfers happened between plastid-containing organisms and the ancestral animal.

Each of the 14 gene families identified in our analyses shows a complex evolutionary history that differs from perceived organismal relationships in three domains of life. In many cases, multiple eukaryotic sequences are sporadically clustered within bacterial homologs. Such a complex evolutionary history for individual gene (or gene family) is somewhat expected, considering frequent HGT events both within domains (e.g., bacteria and archaea) [25,26] and between domains (e.g., bacteria or archaea to unicellular eukaryotes) [27-29], EGT following primary and higher level endosymbioses [3,30], and differential genes losses or replacements [31,32] etc. The dynamic occurrence of these factors over evolutionary time may further compound their effects. Therefore, aside from potential phylogenetic artifacts (see next paragraph), the monophyletic relationship between algal and animal sequences observed in this study can largely be explained by two alternative scenarios: (i) gene losses in most protists and fungi and (ii) gene acquisition by animals. For example, genes encoding UDP galactopyranose mutase are restricted to *C. intestinalis*, algae, and bacteria (including cyanobacteria). Phylogenetic analyses show that *Ciona* UDP galactopyranose mutase sequences group with homologs from multiple algae (Additional file 2). This may be attributed to either extensive gene losses from other eukaryotes or unique gene acquisition by *Ciona.* The scenario of extensive gene losses from other eukaryotes, though theoretically possible, is not parsimonious [6]. Gene acquisition followed by duplication appears to be a more plausible explanation for the presence of this gene family in *Ciona*. Other phylogenetic trees provide similar topologies, where *Ciona* sequences form monophyletic groups with homologs from algae and other animals (Figures 1,2 and 3; Additional file 2). For some identified gene families, other lines of evidence also argue strongly against the scenario of differential gene losses in other eukaryotes; such evidence includes 1) the presence of identifiable homologs only in animals and plastid-containing eukaryotes; 2) the monophyly of animal sequences within algal homologs; 3) uniquely shared domain structures between animal and algal sequences e.g. (Figure 3); 4) the availability of a large number of fungal genomes and the absence of some identified genes in fungi. Furthermore, two of the 14 gene families show cyanobacterial/plastidic affiliation (Figures 1A and 2)A, suggesting likely algal origin. Several other gene families also encode proteins that are targeted to chloroplasts in green plants (e.g., *Arabidopsis thalianaChlamydomonas reinhardtii* and *Micromonas pusilla*) (Additional file 1).

Accurate identification of HGT may be affected by multiple factors. Improper methodology, poor data quality, and insufficient or biased taxonomic sampling are among the factors leading to incorrect gene tree topologies [33]. Especially, long-branch attraction, which may occur when two sequences evolved much faster or slower than others, demands particular attention [34]. In order to reduce the potential complications resulting from these issues, we searched the NCBI non-redundant (*nr*) protein sequence database, ESTs and other sequence data to ensure a broad sequence sampling. We further examined the data quality and performed careful phylogenetic analyses by selecting the optimal substitution matrix for each dataset and using different computational algorithms. Nevertheless, with improved phylogenetic algorithms and more sequence data from other eukaryotes becoming available, we expect that the tree topologies for the identified genes may vary. It should also be noted that, because the phylogenetic approach tends to underestimate the number of acquired genes and because the *Ciona* genome was the sole query in our analyses, it is likely that other algae-related genes may exist in animals. Therefore, the identified genes in our analyses should not be considered as a definitive list of algae-related genes in animals.

### Algal genes and the historical distribution of plastids

Given their presence and relatedness in multiple animal groups, the 14 genes families identified in this study, if indeed of algal origin (algal genes hereafter), most likely were acquired by the ancestral animal. This ancestral animal was probably unicellular, like its extant closest relatives choanflagellates [22]. The identified gene families might have been acquired through either symbioses or feeding activities. In the first scenario, the ancestral animal acquired algal genes as a result of endosymbioses with algal cells, followed by loss of plastids. The second scenario suggests that algal genes were routed to the nucleus of the ancestral animal after algal cells were phagocytosed. In most symbioses between algae and animals, algal symbionts either form loose extracellular association with animals or are sequestered in vacuoles of host cells [35]. Thus far, neither scenario is known to lead to gene transfers. Corals and salamander *Ambystoma maculatum*, for example, enjoy an endosymbiotic relationship with the dinoflagellate *Symbiodinium* and the green alga *Oophila amblystomatis*, respectively [36,37]. But neither EGT between corals and dinoflagellates nor between salamanders and green algal endosymbionts has been reported. By far, only the endosymbioses in sea slug *Elysia chlorotica* and cnidarian *Hydra viridis* are reportedly accompanied by transfer of algal genes [35,38-40]. Because such endosymbioses-mediated gene transfer is unknown in other animals, it might be extremely rare. Given the number of algal gene families identified in our study and their miscellaneous donor groups (Table 1; Figures 1,2 and 3; Additional file 2), it is highly improbable that these gene families are derived from such rare events. On the other hand, because most of the algal gene families identified in animals are derived from microscopic phytoplanktons that are widely distributed in aquatic environments, it is plausible that the ancestral animal obtained these algal genes via food capture.

The algal gene families identified in animals and their possible acquisition from food sources bear important implications for interpreting the historical distribution of plastids and eukaryotic photosynthesis. The origin of plastids is one of the most important evolutionary events in eukaryotic evolution. However, the historical distribution of plastids and photosynthesis in eukaryotes is still heatedly debated [14,16-18,41,42]. Because of the potential loss of plastids, algal genes in aplastidic eukaryotes have often been interpreted as derived from historical plastids. For example, algal genes in apicomplexan *Cryptosporidium*, heterokont *Phytophthora*, and ciliates have been cited as evidence for plastid losses in these taxa [14,33,43]. Similar arguments were also made for kinetoplastids based on the presence of “plant-like” genes [44], though whether these genes are indeed of plant origin is questionable [45,46]. Historical existence of plastids was also suggested for some protozoan species such as heterolobosean *Naegleria*[15,47]. Although endosymbiosis indeed appears to be a reasonable explanation for the observed data in some cases, recent findings of widespread HGT events in eukaryotes raise serious questions about whether all algal genes are derived from historical algal endosymbionts or plastids.

Many studies show that HGT from bacteria is frequent in unicellular eukaryotes [28,29,48]. There is also convincing evidence for HGT between eukaryotes [4,49-51]. In particular, recent studies identified over 100 algal genes in the choanoflagellate *Monosiga*[19,21]. The number of algal genes in *Monosiga* is considerably greater than those in many other aplastidic eukaryotes (e.g., ciliates, *CryptosporidiumNaegleria* etc) that have been suggested to contain historical plastids. Although the possibility that algal genes in *Monosiga* are derived from an obsolete algal endosymbiont or plastid cannot be confidently excluded, the phagotrophic lifestyle of *Monosiga* points to the possibility that these algal genes are derived from food sources. Considering that genes from other prokaryotes can be horizontally acquired by eukaryotes, it should not be surprising to see cyanobacterial (or plastidic) genes in aplastidic eukaryotes. Likewise, it should not be expected that algal genes are immune to HGT, considering that HGT events do occur between other eukaryotes. This may be particularly true for phagotrophic organisms feeding on miscellaneous microbes including unicellular algae. In such cases, algal genes in aplastidic eukaryotes may not necessarily represent relics of historical plastids. These genes should be treated equally as other HGT-derived genes when they are used to infer historical endosymbionts [41], unless other independent evidence (e.g. plastid existence in closely related taxa and compatible phylogenetic signals from multiple genes) is available. Our finding of algal genes in animals provides additional evidence about the distribution of algal genes in aplastidic eukaryotes. Additionally it is consistent with the earlier suggestion that algal genes are expected in phagotrophic eukaryotes or descendants of phagotrophic ancestors [21].

### Evolutionary significance of algal genes in animals

Several cases of HGT between bacterial endosymbionts and their animal hosts have been documented [52-55]. Moreover, HGT from other free-living organisms has also been reported in cnidarians [39,56,57], bdelloid rotifers [58], insects [59], and nematodes [60-62] and other animals [63]. The acquired genes are often linked to novel phenotypes and abilities in recipient organisms, such as improved preying capability in cnidarians [56] and reduced susceptibility to predation in aphids [59]. These HGT cases predominantly occurred more recently in taxa of lower taxonomic ranks and, therefore, do not affect the evolution of entire animal lineage.

Despite the increasing number of HGT-derived genes reported in animals, a common belief is that HGT in animals is rare and its role is limited. This belief largely stems from the fact that the acquired genes need to overcome the germline barrier in order to be transmitted to next generations [4,5]. However, such a germline barrier should not exist in the unicellular ancestor of animals and it is conceivable that the ancestral animal, like its closest relatives choanoflagellates or many other unicellular eukaryotes [29,64], was subject to more frequent HGT. In our study, 13 of the 14 gene families identified in *Ciona* are most likely derived from ancient HGT events. These anciently acquired genes have been vertically inherited, duplicated and retained in diverse groups of extant animals, and thus have contributed to the long-term evolution of animals. Such frequent duplication following gene acquisition has also been observed in other studies [62,65-68] and, in many cases, associated with positive selection in recipient organisms [66,67].

Compared to *Monosiga*, the algal gene families identified in animals are considerably fewer and differ distinctly in their functions. Given the limited number of choanoflagellate genomes available, it is unclear what percentage of the identified algal gene families in *Monosiga* were anciently acquired. However, the lower number of anciently acquired genes in animals is consistent with the suggestion that most acquired genes will eventually be deleted from the recipient genome over time [69]. While the algal genes in *Monosiga* are predominantly involved in carbohydrate and amino acid metabolism [21], five of the 14 gene families, which account for 80% (74/92) of algal genes identified in *Ciona*, are functionally related to molecule transport. The vast majority of these transporter genes (72/74) belong to four solute carrier (SLC) families, including SLC6, SLC22, SLC23 and SLC34 (Table 1), which include transporters of neurotransmitter, organic cation, zwitterion/cation, organic anion, Na^+^−dependent vitamin C transport and phosphate ion, participating in uptake or excretion of numerous important compounds [70-73]. Besides these SLC families, another identified algal gene family is involved in potassium ion transport [74] (Table 1). As these genes encode multiple membrane-bound transporters, substrates such as amino acids, oligopeptides, sugars, inorganic cations and anions, essential metals, biogenic amines, vitamins, nucleosides and ammonia can be transferred across membranes, which in turn accelerates intercellular communication and leads to more efficient cellular metabolism. Several other acquired algal gene families in animals encode proteins that are functionally related to signaling and hormone biosynthesis. For example, the large short-chain dehydrogenase/reductase family (SDR) (Table 1) encodes enzymes involved in cellular differentiation and signaling [75]. Additionally, the biphenyl/valacyclovir hydrolase family is related to aromatic compound metabolism, which may allow the ancestral animal to access diverse digestible food sources.

As all functional information for the algal gene families identified in our analyses is based on investigations of homologs in humans and model organisms, whether these genes have exactly identical functions in other animals remains to be further studied. However, considering the high percent identifies (30%-80%) and close affinities between homologs from humans and other animals (Figures 1,2, and 3, Additional file 1), it is likely that these genes have similar primary functions. Even if the identified genes might have different functions in other animals, they could provide raw stocks for gene and functional differentiation. Extensive intercellular communication is a major feature distinguishing animals from unicellular eukaryotes, and such communication requires proteins related to molecule transport and signal transduction in multicellular animals [76,77]. The acquisition of genes related to molecular transport and signaling and their subsequent duplication might have facilitated the gene renovation and multicellular development in animals. Therefore, our findings, although based solely on analyses of algal genes in a relative small genome of *Ciona*, point to an important contribution of HGT to animal evolution.

## Conclusions

Phylogenomic analyses of the tunicate *C. intestinalis* provide evidences for the existence of algal genes in animals. However, the existence of algal genes in animals does not necessarily constitute evidence for historical occurrence of plastids. Almost all algal gene families identified in our analyses were likely transferred to the ancestral animal and duplicated afterward. Most identified algal genes are related to molecule transport and signaling, suggesting their important role in intercellular communication and possibly the origin of multicellularity in animals.

## Methods

### Data sources and genome screening

The annotated genome of the tunicate *C. intestinalis* was downloaded from the Joint Genome Institute (http://genome.jgi-psf.org/Cioin2/Cioin2.home.html). Expressed sequence tags (ESTs) of 50 diverse eukaryotes (Additional file 3) were downloaded from the NCBI dbEST database and the Taxonomically Broad EST Database (TBestDB) [78]. Assembly of these ESTs was carried out using CAP3 [79]. The resulting consensus sequences were translated in all six frames using *transeq* of the EMBOSS package [80]. All other sequences used in the analyses were retrieved from the NCBI *nr* protein sequence database.

A comprehensive database was created by including the *nr* database and other available eukaryotic genomes (Additional file 3). Genome screening for candidates of HGT-derived genes was performed using *AlienG*[23]. *AlienG* presumes that sequence similarity is correlated with sequence relatedness and it identifies candidates of acquired genes by comparing query sequence similarity to homologs from potential donors and those from closely related taxa. Genes with identifiable homologs only in potential donors are also identified by *AlienG*. In this study, candidate genes of algal origin in *C. intestinalis* were obtained if they showed significantly higher sequence similarity to algal or cyanobacterial homologs than to those from other eukaryotes; the significant sequence similarity to algal homologs was empirically set to a bit score ratio of over 1.5.

### Phylogenetic analyses

For each gene candidate predicted by *AlienG*, we performed further detailed analyses of taxonomic distribution, gene structure, and molecular phylogeny. Protein sequences were sampled from representative groups within each domain of life (bacteria, archaea, and eukaryotes) based on the blastp results against *nr* database. To ensure that sequences were sampled as broadly as possible, we also retrieved sequences from other available eukaryotic genomes and EST databases (see above section).

Multiple protein sequence alignments were performed using ClustalX [81,82] under the default settings, followed by manual refinement. Misaligned sequences, gaps and ambiguous sites were removed manually. The alignment data are available upon request. Phylogenetic analyses were performed with a maximum likelihood method using PhyML 3.0 [83] and a distance method using PHYLIP 3.69 [84]. ModelGenerator [85] was used to select the available model of protein substitution and rate heterogeneity that best fit each dataset. Bootstrap analyses with 100 pseudoreplicates were performed in both methods. Programs from the PHYLIP 3.69 package were used to create pseudoreplicate datasets (SEQBOOT), compute distance matrix (PROTDIST), calculate distance trees (NEIGHBOR), and generate the bootstrap consensus tree (CONSENSE). Gene trees were depicted by combining phylogenies from PhyML 3.0 and PHYLIP 3.69 using TreeGraph 2 [86,87].

## Competing interests

The authors declare that they have no competing interests.

## Authors’ contributions

JFW and JH conceived and designed the study and wrote manuscript. TN performed the analyses and wrote the manuscript. JY participated in the data interpretation and wrote the manuscript. GLS and YZ contributed to the data generation. All authors read and approved the final manuscript.

## Supplementary Material

Additional file 1 **Table S1 and Table S2.** Gene identifiers, chromosomal locations and functions for algal gene families identified in this study. Click here for file

Additional file 2 **Figure S1-S8.** Molecular phylogenies of algal genes identified in *Ciona intestinalis.* Click here for file

Additional file 3 **Document.** List of 50 eukaryotes whose ESTs were used and other 14 complete genome sequences were used in this study, in addition to the NCBI *nr* database. Click here for file
