# Supplementary material for: Ancient gene transfer from algae to animals: Mechanisms and evolutionary significance
Source: BMC Evol Biol. 2012 Jun 12;12:83. doi: 10.1186/1471-2148-12-83 (PMC3494510; doi:10.1186/1471-2148-12-83)
Supplement: Additional file 2 — Figure S1-S8. Molecular phylogenies of algal genes identified in Ciona intestinalis. [file 1471-2148-12-83-S2.pdf]

Molecular phylogenies of algal genes identified in *Ciona intestinalis*. Numbers above branches show bootstrap support values inferred from maximum likelihood and distance analyses, respectively. Asterisks indicate values lower than 50%. Other bootstrap values below 50% in both methods were not shown. Red: tunicates; green: Plantae; blue: cyanobacteria; pink: other plastid-bearing eukaryotes. The alignment data are available on request.

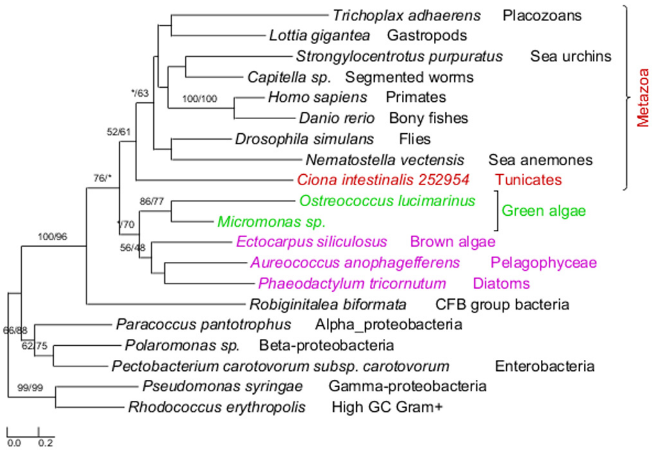

Figure S1. Molecular phylogeny of aminoglycoside phosphotransferase.

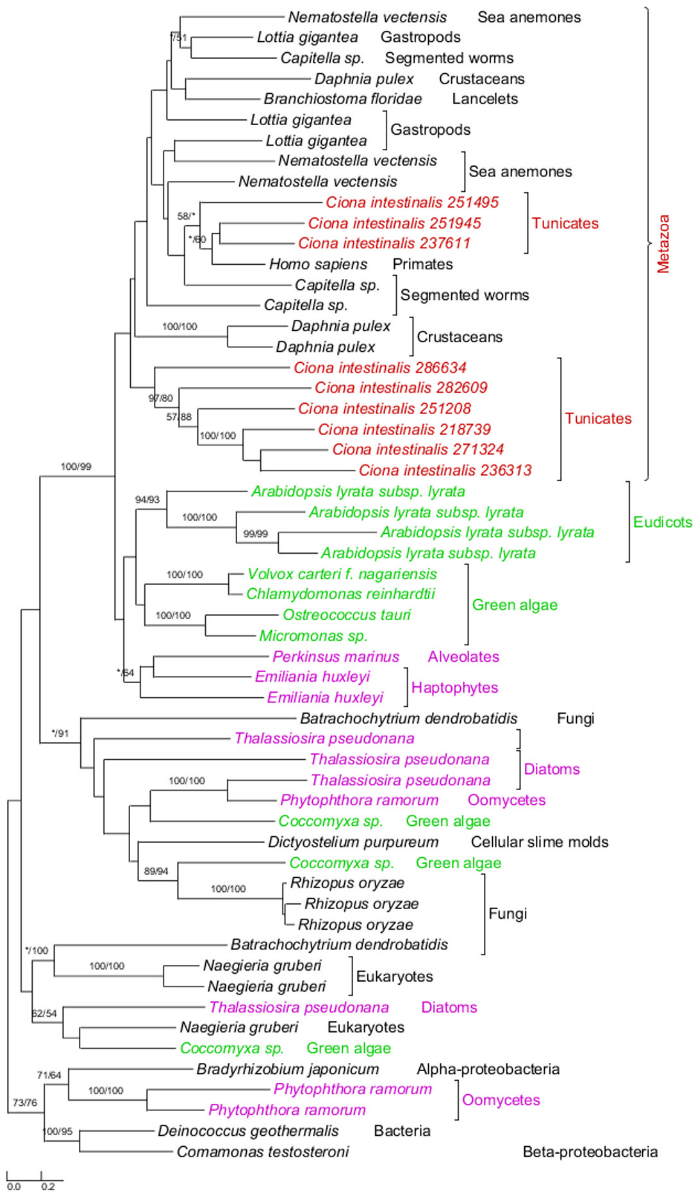

Figure S2. Molecular phylogeny of solute carrier family 22. Only nine of the 27 *Ciona* genes belonging to this subfamily are included here. Additional analyses indicated that all the 27 *Ciona* genes form a monophyletic group with other animal sequences.

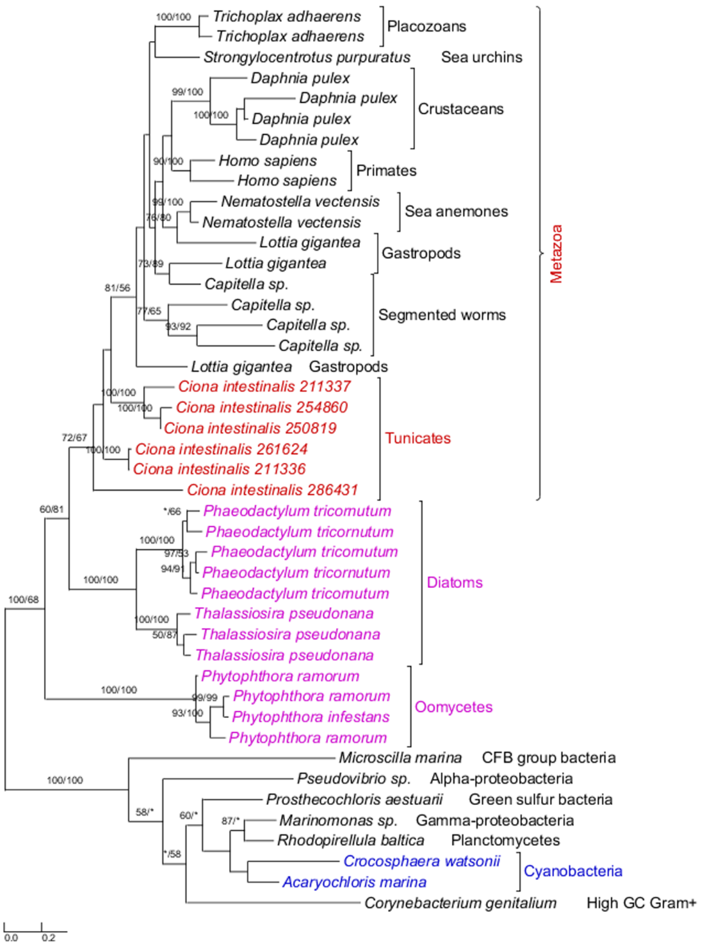

Figure S3. Molecular phylogeny of solute carrier family 34.

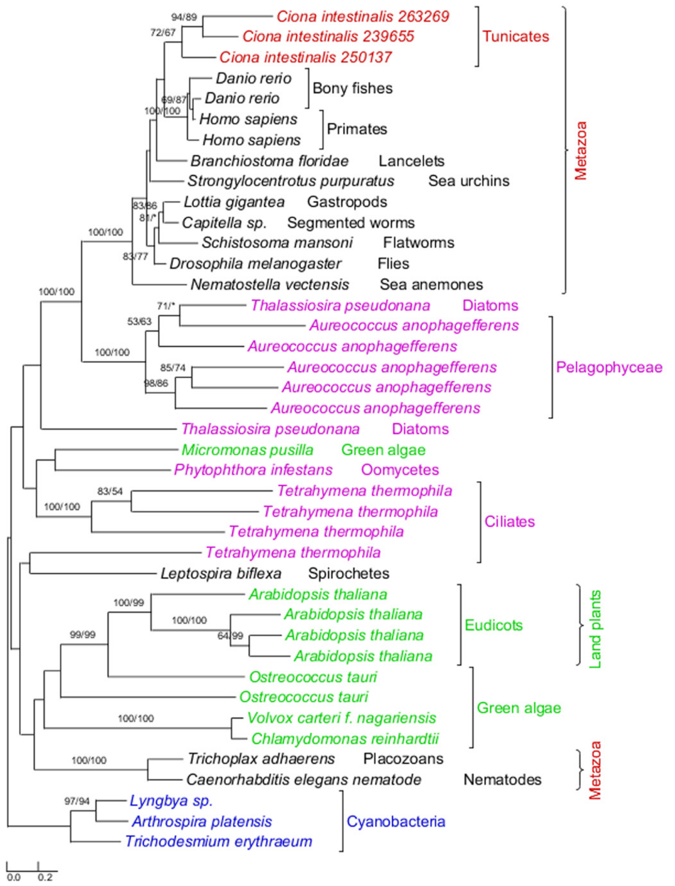

Figure S4. Molecular phylogeny of potassium/sodium hyperpolarization-activated cyclic nucleotide-gated channel 2.

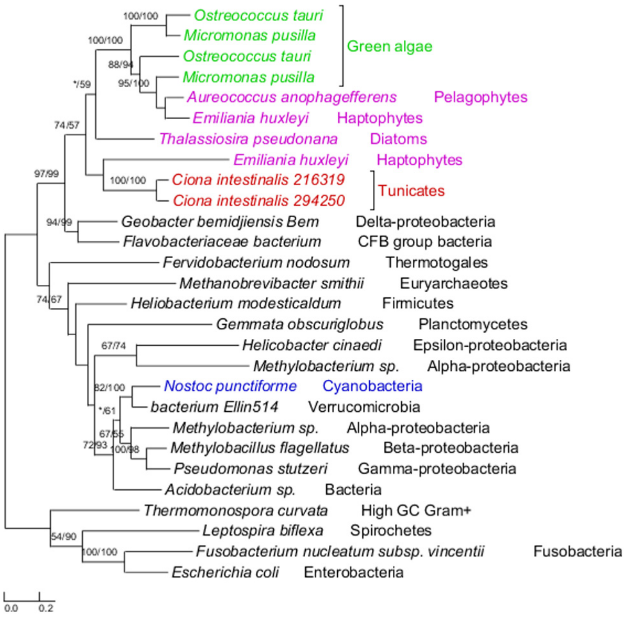

Figure S5. Molecular phylogeny of UDP galactopyranose mutase.

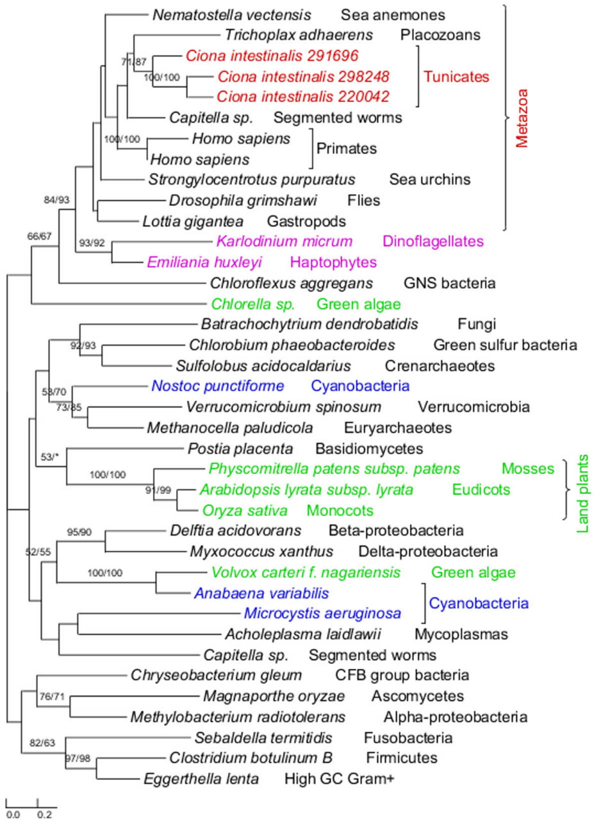

Figure S6. Molecular phylogeny of biphenyl/valacyclovir hydrolase.

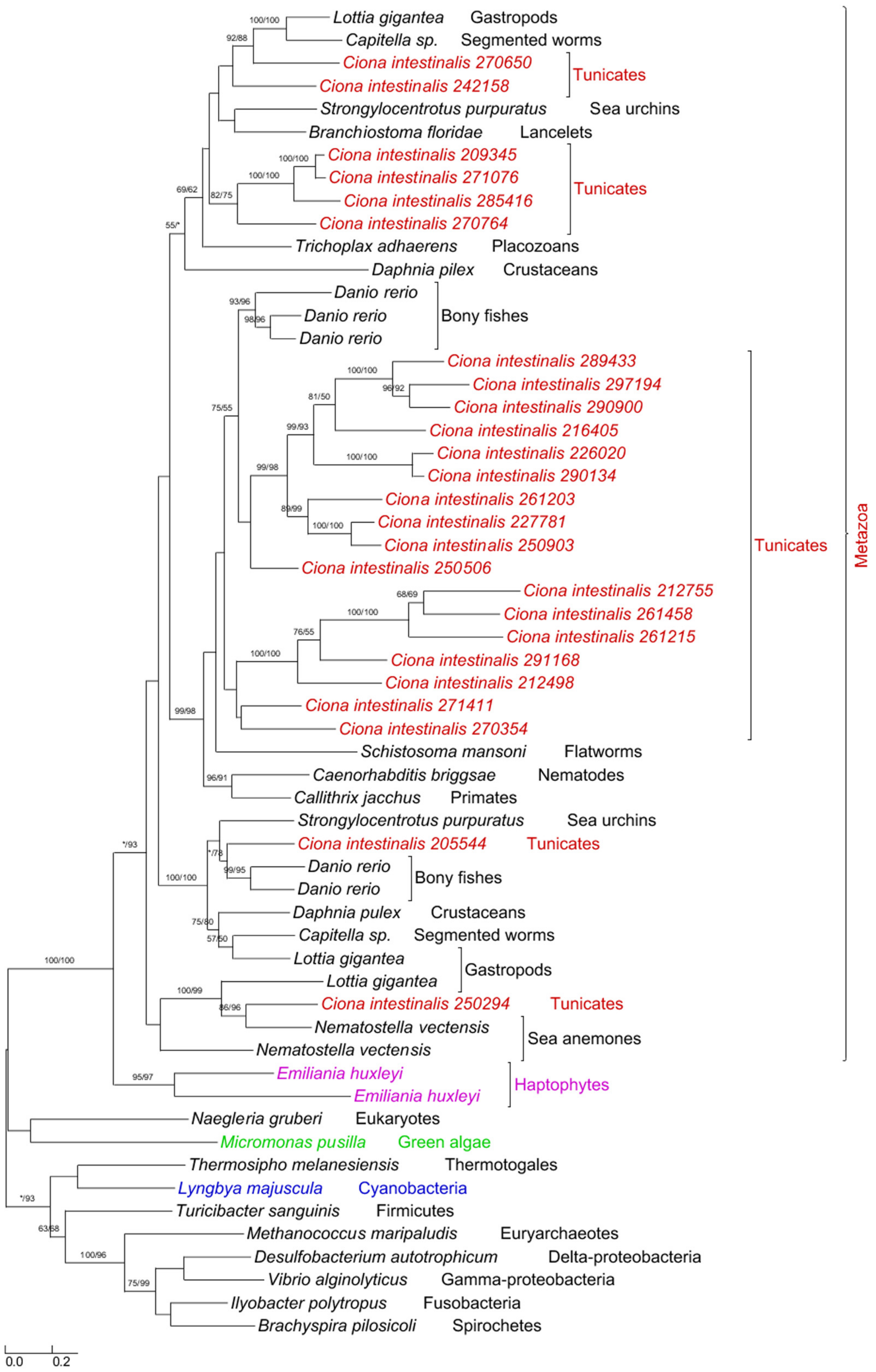

Figure S7. Molecular phylogeny of solute carrier family 6. Only 25 of the 31 *Ciona* genes belonging to this subfamily are included here. The missing 6 gens have extremely high similarity with some of the included genes.

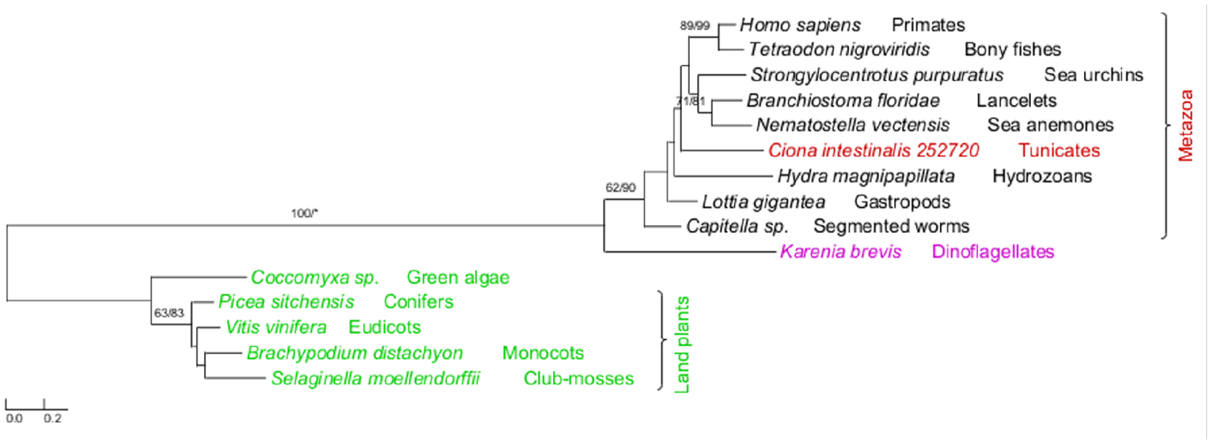

Figure S8. Molecular phylogeny of alkylation repair homolog 5
