# Supplementary material for: Ancient gene transfer from algae to animals: Mechanisms and evolutionary significance
Source: BMC Evol Biol. 2012 Jun 12;12:83. doi: 10.1186/1471-2148-12-83 (PMC3494510; doi:10.1186/1471-2148-12-83)
Supplement: Additional file 3 — Document. List of 50 eukaryotes whose ESTs were used and other 14 complete genome sequences were used in this study, in addition to the NCBI nr database. [file 1471-2148-12-83-S3.doc]

**List of 50 eukaryotes whose ESTs were used and other 14 complete genome sequences were used in this study, in addition to the NCBI *nr* database.**

50 diverse eukaryotes whose ESTs are used:

*Euglena gracilis*

*Physarum polycephalum*

*Acanthamoeba castellanii*

*Spironucleus vortens*

*Cyanophora paradox*

*Blastocystis hominis*

*Porphyra yezoensis*

*Heterocapsa triquetra*

*Paracercomonas marina*

*Monosiga ovata*

*Malawimonas californiana*

*Karlodinium micrum*

*Astasia longa*

*Proterospongia sp*

*Diplonema papillatum*

*Seculamonas ecuadoriensis*

*Polytomella parva*

*Acetabularia acetabulum*

*Hyperamoeba dachnaya*

*Amphidinium carterae*

*Karenia brevis*

*Prototheca wickerhamii*

*Emiliania huxleyi CCMP1516*

*Capsaspora owczarzaki*

*Helicosporidium sp*

*Sphaeroforma arctica*

*Oxytricha trifallax*

*Isochrysis galbana*

*Alexandrium tamarense*

*Hartmannella vermiformis*

*Mastigamoeba balamuthi*

*Pavlova lutheri*

*Glaucocystis nostochinearum*

*Streblomastix strix*

*Sawyeria marylandensis*

*Antonospora locustae*

*Trimastix pyriformis*

*Scenedesmus obliquus*

*Guillardia theta*

*Stachyamoeba lipophora*

*Malawimonas jakobiformis*

*Reclinomonas americana*

*Jakoba libera*

*Bigelowiella natans*

*Mesostigma viride*

*Hyperamoeba sp*

*Histiona aroides*

*Brachionus plicatilis*

*Polysphondylium pallidum*

*Jakoba bahamiensis*

Other 14 eukaryotes with available genomes:

*Aureococcus anophagefferens*

*Rhizopus oryzae*

*Phytophthora ramorum*

*Lottia gigantea*

*Cyanidioschyzon merolae*

*Coccomyxa sp.C-169*

*Chlorella sp.*

*Dictyostelium purpureum*

*Emiliania huxleyi*

*Thalassiosira pseudonana*

*Batrachochytrium dendrobatidis*

*Capitella sp.*

*Naegleria gruberi*

*Phytophthora sojae*
